# Hatchling survival to breeding age in Northern Pine Snakes (*Pituophis melanoleucus*) in the New Jersey Pine Barrens: Human effects on recruitment from 1986 to 2017

**DOI:** 10.1371/journal.pone.0195676

**Published:** 2018-05-14

**Authors:** Joanna Burger, Robert T. Zappalorti, Michael Gochfeld

**Affiliations:** 1 Division of Life Sciences, Rutgers University, Piscataway, New Jersey, United States of America; 2 Herpetological Associates, Inc. Pemberton, New Jersey, United States of America; University of South Carolina, UNITED STATES

## Abstract

To conserve threatened/endangered species, we need to understand the factors contributing to reproductive success and recruitment to reproductive stage. Obtaining this information is difficult for snakes because they are secretive, are not easy to locate at the same stage each year, and are sometimes sparsely distributed. We determined nest fate, hatchling growth and survival to age 5 years, and recruitment to breeding age of Northern Pine Snakes (*Pituophis melanoleucus*) in New Jersey Pine Barrens from 1986 to 2017. Pine Snakes are ‘threatened’ in New Jersey and in other states, and are at risk because of increased human population, habitat loss, predation, and poaching. Age of first-breeding was 4-years, based on snout-vent length of gravid and laying females, and snout-vent length of females followed as hatchlings to 5-years. Mean clutch size (+ 1 SE) was 9.5 + 0.3 (N = 53). The annual percent of nests in which eggs hatched averaged 25% (N = 288 nests), and varied among 5-year periods (5% to 30%/year). Of lab-reared hatchlings released into natal nests (N = 90), 26% (2015) and 32% (2016) reached hibernacula excavated in 2016 and 2017. The sex ratio of hatchlings reaching hibernation sites (N = 181) between 1986 and 2015 was skewed toward females (74/106, 59% females), and varied among 5-year periods (47–75% females). Once hatchlings reached a hibernaculum, there was a sex-related difference in survival. For hatchlings reaching a monitored hibernaculum, survival to 3-years was 35% in females and 40% in males, and to 4-years was 25% in females and 33% in males. Using these data, only 10% of females reached 3 years (first possible breeding age), and 7% survived to 4-years. Methodological problems with determining survival rates during these early critical years are discussed.

## Introduction

Demographic and life history data provide the information necessary for conservation efforts, and are particularly critical for threatened or endangered species, and species whose populations are declining or their habitats are imperiled. Population stability is largely a function of survival and reproduction [1]. One of the fundamental factors determining population dynamics and viability is recruitment to breeding age, which in turn is related to female age of first breeding, birth or hatching rates, offspring growth, and offspring survival [2, 3]. These factors are influenced by genotype, maternal effects, environment, colonization history, latitude, gender, or a combination of these [2, 4–7]. Because of variations in these factors, long-term studies for all aspects of snake ecology are essential [8]. Such long-term studies are also essential because life history traits often co-vary [9].

Presumably, current investment in reproduction may have a deleterious effect on future reproduction, giving rise to a conflict between current reproductive effort (parental investment) and survival to reproduce again in future years [9, 10]. Costs arise when a female invests energy in current reproduction rather than in growth, decreasing future fecundity [10]. Costs for females involve not only production of eggs in the case of reptiles and birds, but costs associated with nest site selection, nest building, nest defense, and/or incubation and defense of young (the latter two occur in birds). In addition, costs of reproduction increase if individuals are more vulnerable to predators or starvation while engaged in reproduction [11]. Water snakes (*Nerodia sipedon)*, for example, experience some decreased winter survival of reproductive females related to their emaciated state following parturition [11].

Species with indeterminate growth (e.g. many fishes, snakes) exhibit large variation in life history traits [3, 12, 13]. Determining demographic and life history traits for long-lived snakes is difficult because most species are often secretive, occur in low numbers, have long periods of inactivity, are unpredictable, and cannot be captured reliably every year [3, 14, 15]. Few long-term studies of snakes exist [1, 16–19]. Capture probability also varies by season in snakes [15], making it difficult to examine demographics and recruitment. Understanding the demographics and population dynamics of snakes is important because they can be key components of ecosystems in terms of local biomass, as predators, and because many are threatened or endangered [15, 18–21].

Size and age at maturity for snakes (e.g., reproductive age) are major life history traits because they influence lifetime fecundity [3]. Age at maturity is a critical aspect of life history strategies because fitness is sensitive to changes in this trait [3]. In general, body size at maturation is a relatively constant proportion of maximum size, and adult survival is proportional to age at maturity in snakes and lizards [22]. Squamate reptiles exhibit interspecific and intraspecific variations of growth, survival rate, and maturation [22]. Size (not age) is the critical determinant of reproductive maturity in some snakes [23, 24], and maternal body size relates to litter size, rather than offspring size [9]. Fast juvenile growth leads to early maturation and large body size, which in turn increases early reproduction (which might enhance total reproductive lifespan [16]).

Determining juvenile growth is difficult because most snakes are not easy to find as hatchlings, or at the same life-stage each year. It is also difficult to determine nest success, hatchling survival to reach a hibernaculum, and survival to reproductive age in snakes, especially Northern Pine Snakes (*Pituophis melanoleucus*) because they spend a great deal of time below ground in summer dens or other burrows, under leaf litter, or in rotting logs [18]. We were able to locate traditional nesting and hibernating sites that have been used for many years in the New Jersey Pine Barrens. Female Pine Snakes leave a recognizable dump pile of sand during excavation of nests [25–28]. Pine Snakes are the only North American snake to regularly excavate their own nesting burrow, tunnel and egg chamber, allowing us to locate nests, often with gravid females or those that have just laid their eggs [29].

The specific objective of the present study is to use long-term field data collected from 1986 to 2017 to examine survival and growth to breeding age (recruitment into the breeding population). Our overall long-term goal is to understand the factors that contribute to growth rates, survival, reproduction, habitat selection, fidelity, behavior and longevity of Northern Pine Snakes in the New Jersey Pine Barrens, and to determine which factors are amenable to management.

To determine recruitment of Northern Pine Snakes into the breeding population, the following parameters need to be known: average age of first breeding, clutch size, nest success, hatching rate, growth rate, and survival to first breeding age. While some of these can be determined in captivity, it is necessary to have data from free ranging snakes in the wild. The present study examines survival and growth to reproductive age of Pine Snakes from 1986 to 2017. We report on the percent of nests that successfully hatch, the percent of young that reach a monitored hibernaculum, the percent of young that reach 5-years of age, and the growth patterns of snakes from hatching to 5-years. Of particular interest were temporal differences in these traits over the 30-year period, as well as the effect of human activities (poaching, off-road-vehicles) and management to prevent these causes of mortality. Data are also presented on age of first reproduction in females based on snout-vent length of gravid and laying females in nature. The data used are from known-aged snakes located as hatchlings in hibernacula, at about 5–7 months post-hatching.

## Methods and protocol

### Ethics statement

Field and laboratory research in this study are approved by the Rutgers University Institutional Animal Care and Use Committee (Protocol 86–017; active from 1986 to the present, with 3-year renewals). All necessary permits are obtained annually from the New Jersey Department of Environmental Protection (Endangered and Nongame Species Program), and this research is conducted with permission from land-owners. In all cases, the health and welfare of the snakes comes first.

### Study species, study sites, and age classification

Northern Pine Snakes are large constrictors that reach the northern limit of their range in the New Jersey Pine Barrens. They are among the top-level predators in the region and can grow to almost 2-meters long [28]. *Pituophis melanoleucus* has three subspecies: the Florida Pine Snake (*P*.*m*. *mugitus*), the Black Pine Snake (*P*. *m*. *lodingi*) that is federally threatened [30], and the Northern Pine Snake (*P*.*m*. *melanoleucus*) that is threatened in New Jersey. Some lines of molecular evidence suggest that these three are part of a trans-continental multispecies complex [31]. The New Jersey population of Northern Pine Snakes is isolated from other populations living to the south by several hundred km [18, 19, 29]. This species is declining in many parts of its range, and is not common anywhere. The declines of the species to the south, and its threatened status in New Jersey, make it imperative to understand the factors impacting population levels. Hereafter we are referring only to the Northern Pine Snake, unless otherwise noted.

Pine Snakes in the New Jersey Pine Barrens excavate their own nest in open-canopy sandy areas, showing high fidelity to these exact nest sites. Sometimes several females lay eggs in the same nest [32, 33]. Excavation can take several days, and digging females normally rest during the hottest part of the day in the shade of pine trees. Nesting females and their nests are vulnerable to off-road vehicles (ORVs), poachers, and predators, as are hatchlings [19, 34–37]. Hatchlings presumably find their way to hibernacula by following adult scent trails [9, 38, 39]. Adults have relatively large territories, and radio-tracked snakes can be found as far as 3–4 km away from hibernation and nesting areas [19, 27, 28]. They spend the winter in communal hibernacula that they modify from old mammal burrows and old stumps, digging long tunnels out into virgin sand and overwintering in chambers they excavate [18, 34, 40, 41].

We study Pine Snakes in Bass River State Forest, on Nature Conservancy property, on Wildlife Management Areas, and on private land. The types and degree of management vary. Nesting and hibernating areas are generally located in relatively-exposed sandy areas dominated by Pitch Pine (*Pinus rigida*) and various oak species (*Quercus* spp.)[29, 42–45]. Exact locations of study sites are not given because of the threat of poaching basking snakes when they first enter or leave hibernacula, and the threat of poaching gravid and nesting females (in some years, 40% of our unmarked, but mapped nests were taken by poachers [36]).

From 1986 to 2017 we conducted field studies on nesting snakes (1 June to 15 September), on hatchlings (late August to early November) and on hibernating snakes (20 February and 17 March). Our marking and recapture methods have not adversely affected their behavior or survival [41, 46].

### Protocol for nesting females

Generally gravid females nest in openings or clearings within Pitch Pine and Blackjack Oak (*Quercus marylandica*) forest uplands, including old farm fields, railroad beds, natural forest clearings, and the shoulders of paved or dirt roads [29, 42]. Known snake nesting areas (N = about 18) were searched from 1976 to the late 1980s for nests that were in or adjacent to hibernacula. Thereafter we selected three primary areas for our work (mainly due to loss of other nesting areas to poaching because they were close to roads or houses where they were easily accessed). Snakes encountered within our study area are captured and searched for markings (brands used in the early 1980s, or PIT tags), marked, and released where found. Nests can be recognized by snake trails in the sand, sand fans, small holes, and diagnostic dump piles opposite the burrow openings [32, 41]. Gravid females are captured as they leave their excavation site in the heat of the day, or a few days after egg laying when they remain in the nest (perhaps guarding eggs). To determine clutch size we later excavate nests, count eggs, and rebuild the nest chamber using glass or clear plastic as a roof to maintain the necessary airspace. The egg mass is left intact (with eggs attached to one another [41]). In some cases 3–4 clutches are laid in the same nest [32]. However, these clutches are distinguishable because females laying eggs exude a liquid, and as it dries it binds the eggs of a clutch together. When a different female enters the same nest tunnel to lay eggs, the previous clutch is already bound together.

We search for nest burrows from mid-June through the first week in July, which is the normal laying period for females. The locations of all nests are marked on maps, rather than with field markers to reduce poaching and predation. Nesting areas are examined periodically during the incubation period to determine if nests are destroyed (or hatched). We assume that nests that are not excavated by predators or poachers have hatched. In some years we excavate them in mid-September to determine hatching success [41] or if snakes failed to emerge from nests (very rare). When eggs hatch, the empty shells have a characteristic slit on the top of the egg.

### Protocol for hibernating snakes

Hibernation sites are discovered by observing basking snakes on the surface near an opening such as a hollowed-out stump hole or abandoned mammal burrow that could represent a hibernacula. These surveys are conducted in the spring and fall seasons, the time of egress in spring and ingress in the fall. When we observe snakes frequently at a potential overwintering site, we excavate the subterranean structure in late winter to search for hibernating snakes [34]. In subsequent years confirmed hibernacula are dug up annually. Snakes are weighed and measured, and hibernacula are reconstructed the same day (we return all snakes into their den on the same day [18]). Protocols and methods for excavation and study of Pine Snakes in hibernacula are described fully in Burger and Zappalorti [18, 26] and Burger et al. [25, 37].

Although we follow all Pine Snakes found in hibernacula, we use only known-aged, marked snakes captured as hatchlings (or as 2-year olds) in studies reported herein. We define their ages as follows: 1 = hatchling found in hibernacula in March (hatched the previous late summer to early fall, about 7 months old), 2 = caught the following year in hibernacula in March, about 1 year and 7 months old), 3 = caught the following year, about 2 years and 7 months old), and so on. All snakes are implanted with PIT tags that each have a 9–10 digit number, allowing quick, easy and accurate identification of each snake [46].

Growth rates were determined for known-aged snakes up until 5 years of age. We determined age of first breeding by comparing the snout-vent length of gravid or laying females with the snout-vent length of known age females determined from snakes marked as hatchlings in hibernation sites. Growth was measured as weight and length at successive ages.

Some hibernacula are used every year (30+ years), while others are not used continually [18, 37]. Black Racers (*Coluber constrictor*) are the only other snake found regularly in these hibernacula, with a few Corn Snakes (*Elaphe (Pantherophis) guttata*), Timber Rattlesnakes (*Crotalus horridus*), Coastal Plain Milk Snakes (*Lampropeltis triangulum*), and Black Rat Snakes (*Elaphe obsoleta*). Many snakes were located in several consecutive years in the same hibernaculum, which indicates that our methods did not unduly distress them. One adult female Pine Snake was located 19 of her 23 years, almost always in the same hibernaculum.

### Experimental determination of hatchlings reaching hibernacula

Starting in 2015, some clutches of eggs were collected from nest burrows and hatched in the laboratory as part of a program to “head start” them because of the low number of hatchlings that reached hibernacula from 2011–2015. From 2011–2015, only 8 hatchlings successfully reached our known hibernacula (down from 23 in the previous 5 years). Clutches were removed to the laboratory and incubated at about 23-25^o^ C, the temperatures found in most wild nests [47]. Once the lab-hatchlings had shed (they often don’t eat before they shed, and usually emerge after shedding [48]), they were weighed, measured, and given a PIT tag. They were then placed in their natal nest burrow, covered with sand so that they were not visible to predators, and allowed to emerge on their own, forage and seek hibernation sites. We searched for them in known hibernacula the following late winter.

### Data analysis

The procedures described above allowed us to determine: 1) clutch size from excavated nests [32], 2) nest success by following nests in the field to determine whether eggs hatched and by digging up nests (identified on maps of nesting sites) that had not been disturbed. 3) percent of hatchlings reaching a hibernaculum by releasing lab-hatched snakes into their original nests, allowing them to emerge naturally, and determining how many reached a known hibernaculum in their first year, 4) growth rates of males and females to age of first breeding by measuring known-aged, wild snakes from hatchling stage to 5 years (captured in hibernacula each March), 5) survival to age 5 by following hatchlings up until 5 years, and 6) age of first breeding. Survival was determined only for snakes found as hatchlings.

Analyses included calculating frequencies and percentages, means and standard deviations, Kruskal-Wallis One Way Analysis of Variance (ANOVA) analysis, and 95% confidence intervals [49]. Kendall tau was used to determine correlations among variables. Means and standard errors are given unless otherwise noted.

## Results

### Clutch size, nest success, and success at reaching a hibernaculum

Mean clutch size (n = 53) was 9.5 + 0.3 (range of 5–14 eggs/clutch), and was positively correlated with snout-vent length of the female (tau = 0.60, P < 0. 001 [32]). Nest hatching success varied by year, and averaged 25%. Nest hatching success was 42% from 1986–1990, 15% from 1991–1995, 8% from 1996–2000, 37% from 2001–2005, 24% from 2006–2010, 30% from 2011 to 2015, and 50% in 2016–2017 (Table 1). The years with low nest success had high ORVs use (churning up nests), which was later prevented by constructing barriers preventing access to nesting areas.

**Table 1:** Fate of nests of Pine Snakes in the New Jersey Pine Barrens, and recruitment of hatchlings to hibernation sites, averaged over 5 year periods (1986–2017). Nest data are from Burger and Zappalorti [18, 19, 32, 41], Burger et al. [36], and unpublished data.

Years	Number of nests	Percent hatched	Mean hatchlings per year	Percent female hatchling	Sex Ratio of hatchling F/M	Conservation issue and management
**1986–1990**	73	42	11.4	47	27/30	Laboratory hatching of clutches increased survival
**1991–1995**	65^a^	15	6.6	67	22/11	Increased ORV activity; ropes across opening to block ORV access (1993)
**1996–2000**	60^a^	8	5.4	70	19/8	Increased ORV activity: small berm + chain in 1996
**2001–2005**	43	37	6.6	64	21/12	Large berm and steel cables in 2001; no ORV activity
**2005–2010**	21	24	4.6	52	12/11	Increased poaching
**2011–2015**	26	30	1.6	75	6/2	Increased poaching
**2016–2017**	8^b^	50	18.5	81	30/7	Laboratory hatching of clutches began in 2015; hatchlings found in hibernation in 2016.
**1986-2015mean (%)**^**c**^	288	30%	6.0	59	107/74	For period 1986–2015

a. Nests based on number expected from previous years nest locations; success based on nests that did hatch (mainly outside area used by ORVs).

b. Fewer nests followed because of a conservation program to lab-hatch clutches to reduce predation and poaching

c. Overall mean does not include 2016–2017 when a high percentage of clutches were taken and lab-hatched to increase hatchling success.

From 1986 to 2015, from 0 to 18 hatchlings/year reached monitored hibernacula, for an overall average of 6 hatchlings per year (Table 1). The percent of hatchlings to reach a hibernacula during the first year ranged from 26% to 32% (N = 90 hatchlings released into their natal nests in 2015 and 2016). This number is conservative because a hatchling may not reach a known hibernation site until it is 2 or more years old. Thus, in 2016 and 2017 the average number of hatchlings in monitored hibernacula was 18.5 due to our release of “head start” hatchlings to increase recruitment (these data were not considered in the nest success calculations.

### Weight and survival rates of male and female hatchlings

From 1986–2015, 59% of the hatchlings found in hibernacula were females, and in every 5-year period (except 1986–1990), female hatchlings outnumbered male hatchlings. In 2015 and 2016, 81% of the hatchlings were females. Survival rates differed between males and females during the first 5 years of life, indicating that females have lower survival rates than males (Table 2).

**Table 2:** Percent survival of male and female Pine Snakes (N of known-aged, hatchlings) until 5 years of age in the New Jersey Pine Barrens. The table is based on 155 female and 91 male hatchlings followed to age 5 years. Shown also is the sex ratio at each age. There were significant survival differences during the first 5 years of life (18.8, P < 0.0001). Given is the percent surviving (sample size).

Age	Females	Males	Sex ratio
**Hatchlings**	100 (N = 155)	100 (N = 91)	0.59
**2-year**	45 (69)	49 (45)	0.65
**3-year**	35 (55)	40 (36)	0.65
**4-year**	25 (38)	33 (30)	0.79
**5-year**	21 (32)	25 (23)	0.72

**Fig 1:**
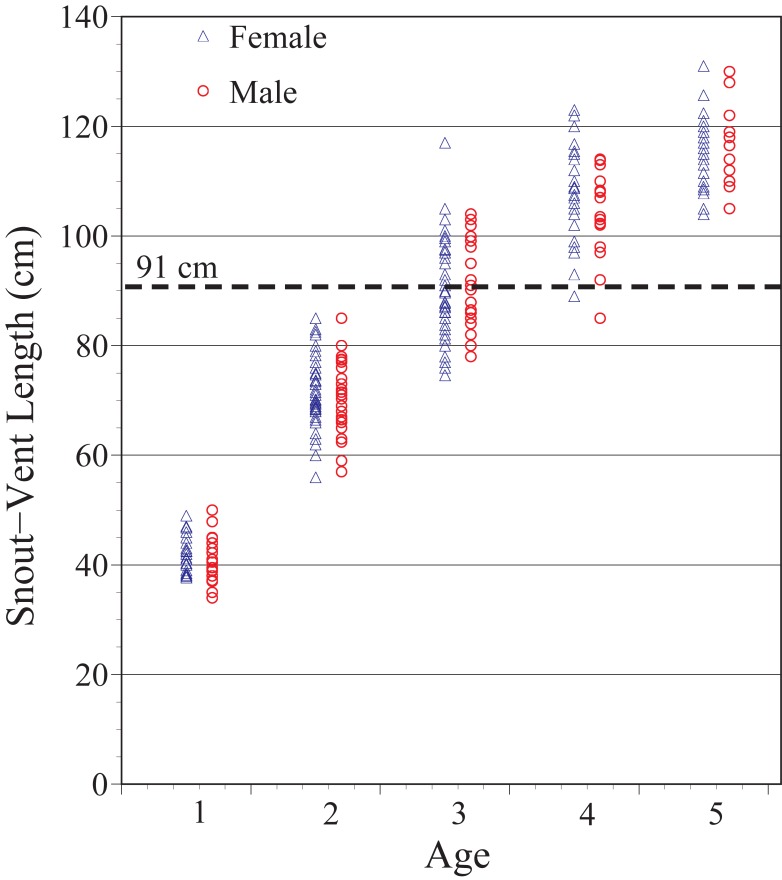
Distribution of snout-vent length of known-aged male and female Pine Snakes in the New Jersey Pine Barrens until age of first reproduction. Single circles or triangles indicate one snake, but the darker the circles, the more overlap in snakes at those sizes. Dotted line = snout-vent length of smallest female known to lay a clutch of eggs.

**Fig 2:**
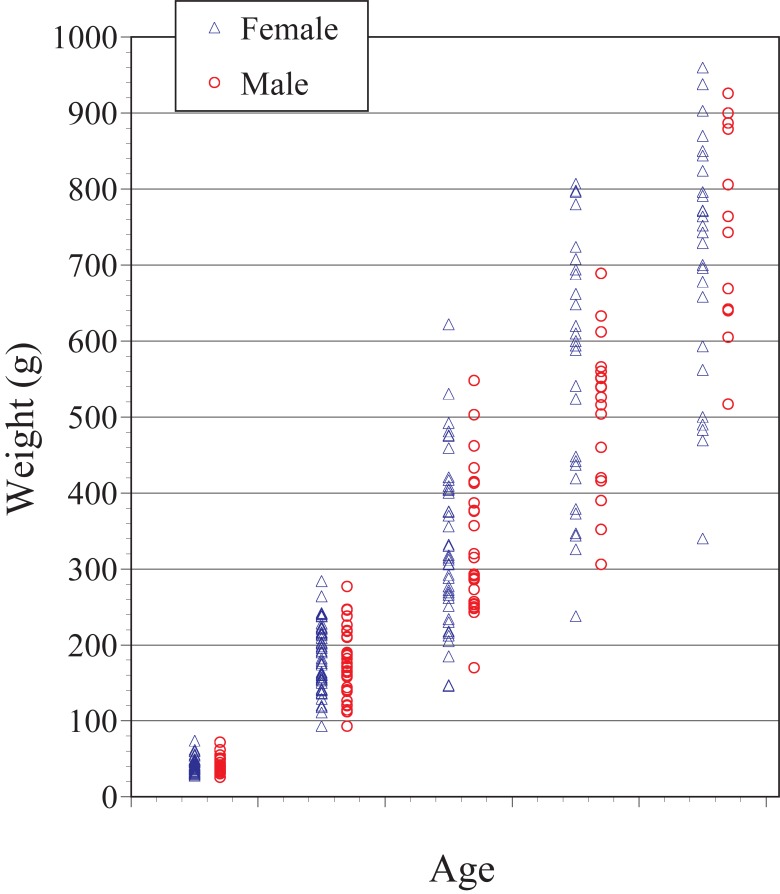
Distribution of weights of known-aged male and female Pine Snakes until age of reproduction. Single circles or triangles indicate one snake, but the darker the circles, the more overlap in snakes at those sizes.

For determining age of first breeding, size (weight and snout-vent length) is a critical component. There was great variation in both weight and snout-vent length for both males and females during the first 5 years of life (Figs 1 and 2), although there were no significant sex-differences in weight or snout-vent length during the first 5 years of life. Both scatter plots (Figs 1 and 2) and means (+ standard deviations) are provided to allow assessment of variation within age classes at the same time of year (March, Table 3). Sample sizes differ because not every snake that survived was found every year. Some snakes were still alive at 5 years (as determined by data from later years), but were not found at 5 years. Thus we included all hatchlings that were alive at 5 years, even if they were not found at age 5 but were found at age 6 or beyond.

**Table 3:** Size of male and female Pine Snakes in the New Jersey Pine Barrens as a function of age. Given are weights (g, means ± standard error) and snout-vent length (cm) for snakes of each age class. No significant differences were detected between males and females in any age class (Kruskal-Wallis test). Raw data is provided in S1 Table.

Age	N	Males			N	Females			X^2^ (p)
**Weight (g)**									
**Hatchling**	65	40.4	±	1.3	126	41.2	±	1.0	0.1 (0.7)
**2-year**	34	177	±	7.5	50	186	±	5.6	0.8 (0.4)
**3-year**	23	336	±	19.8	41	337	±	16.8	0.0 (1.0)
**4-year**	18	507	±	23.5	26	570	±	31.8	2.3 (0.1)
**5-year**	12	748	±	38.7	25	720	±	31.1	0.1 (0.7)
**Snout-vent length (cm)**									
**Hatchling**	66	41.2	±	0.4	126	41.6	±	0.3	0.2 (0.7)
**2-year**	35	70.8	±	1.1	52	72.2	±	0.7	0.4 (0.6)
**3-year**	23	91.0	±	1.7	43	91.1	±	1.4	0.0 (1.0)
**4-year**	19	105	±	1.7	27	108	±	1.7	1.4 (0.2)
**5-year**	12	118	±	2.3	25	116	±	1.2	0.5 (0.5)

**Fig 3:**
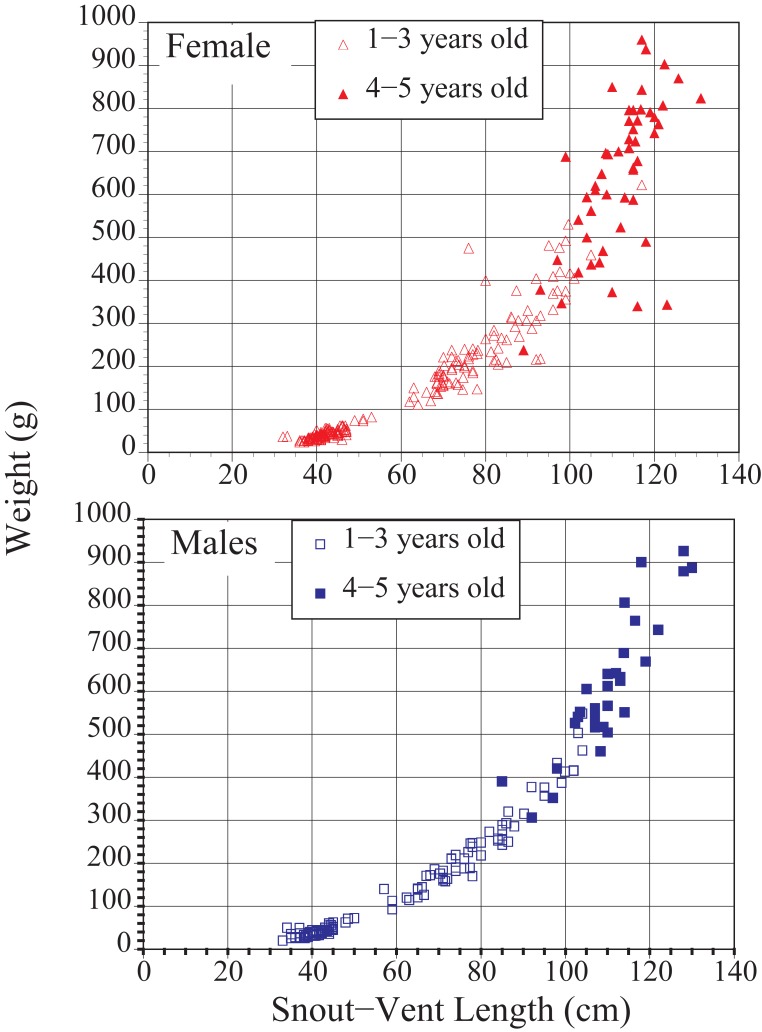
Relationship of weights and snout vent lengths for male and female Pine Snakes from the New Jersey Pine Barrens.

Snout-vent length and weight were highly correlated for males (tau = 0.90, P < 0.0001) and females (tau = 0.87, P < 0.0001 Fig 3). Variation was greater for females than males at 4 and 5 years of age. Three year and four year olds are shown as filled in circles and squares on Fig 3 to show the variances in weight as they enter breeding age.

### Factors affecting weight and survival of hatchling to 5 years of age

Survival to age 4–5 could vary as a function of initial hatchling size (weight, snout-vent length), year of hatching (weather, disturbance), location of hatching (the three study sites), and by maternal factors or environmental conditions (food availability). Hatchlings were divided into three categories for this analysis: hatchlings never found again, hatchlings found at 2 or 3 years of age (but never again), and hatchlings still alive at 4 or 5 years of age. For the latter category a snake found at any later age (e.g. 6, 12, or any other age) was considered alive at age 5 even when it was not caught at age 5. There were no significant differences in survival to age 4–5 as a function of hibernation location, but there were by year (Table 4). Significantly more hatchlings reached 5 years of age in the first three time periods (1986–2000) than in later periods.

**Table 4:** Effect of hatching date on survival of Pine Snakes to age 4–5 years in the New Jersey Pine Barrens. Given is the percent of hatchlings from each 5 year time period in each survival category (X^2^ = 22, P < 0.01).

Time period	Percent of nests in which some eggs hatched	Percent of Hatchlings never found again	Percent of Hatchlings that survived only to 2–3 years of age	Percent of Hatchlings that survived to at least 4–5 years of age
**1985–1990**	42	45	20	35
**1991–1995**	15	72	9	19
**1996–2000**	8	48	26	26
**2001–2005**	37	73	15	12
**2006–2010**	24	92	4	4
**2011–2015**	30	75	25	0^a^

a. This is low because snakes that were hatchlings in 2014 could only have reached 4 years, and those found in 2015 could only have reached 3 years.

There were no differences in the mean hatchling weight (X^2^ = 2.1, P < 0.14), or snout-vent length (X^2^ = 0.005, P < 0.94) for snakes that were only found once (as hatchlings) or as 4 or 5 year olds.

### Age of first breeding

Gravid and laying females located in the field from 1977 to 1989 [32], and 2013 to 2016 indicated that females can lay eggs when snout-vent length is greater than 90 cm (mean = 126 + 14.5 cm, Table 5), which includes some 3 year olds, all but one 4 year old female, and all 5 year olds (dotted line on Fig 1). A scatter diagram (rather than just means + variances) is provided to show the length pattern for all individuals. Although some females in the wild laid eggs when they had snout-vent lengths of 91–100 cm. this does not mean that all females of this size do.

**Table 5:** Number of gravid or laying female Pine Snakes in the New Jersey Pine Barrens that laid eggs in the field, along with their snout-vent lengths (1986–1996, 2013–2016). Thus the shortest female to lay was 91 cm, and the next was 99 cm.

Clutch Size (Number of eggs)	Number of Females to lay this clutch size	Maternal snout-vent length for each laying female (cm)
**5**	1	112
**6**	4	115,120,148,131
**7**	6	109,113, 118, 122, 143,146
**8**	9	99, 112, 118, 122, 143, 142, 146
**9**	14	91, 100, 103, 104, 109, 110,115, 116, 121, 122, 124, 128, 130, 131
**10**	6	119, 120, 129, 130,144,146
**11**	5	115, 122,126, 132,134,
**12**	4	133,135, 144, 147
**13**	1	144
**14**	3	122, 130, 141

## Discussion

The results from this study demonstrate temporal variation over the 30-year period in the percent of Pine Snake nests that hatched, sex ratio of hatchlings, and survival to age 5-years. Temporal differences were related to human activities. There were no significant differences in sizes of males and females up to 5-years, but males had significantly higher survival rates than females (up to 5-years of age). There were no differences in survival and weight gain among the three populations. Some females are capable of breeding at 3 years, and nearly all are by 4-years. These data provide information on recruitment to age of breeding by three populations of Pine Snakes, a highly secretive, long-lived, top-level predator in their ecosystems. Human activities (poaching, ORVs) were a major driver of reduced survival and recruitment in this population. These aspects will be discussed below. As with any long-term field study, there are methodological issues.

### Methodological issues

In our 30 + year study of Pine Snakes, methodological issues involved natural and anthropogenic variations. Some variations may be due to changes in climate and increases in habitat loss (and degradation) or human disturbance. There is some intrinsic variation in snake behavior (e.g. males and female have different movement patterns [27]). Some may be due to our increasing experience over the years (e.g. our search image for nests improved). The period 1980 to 2016 showed a warming trend [50, 51]. With warmer temperatures, snakes may leave hibernation earlier, but it is unlikely we missed them because we dig them up well before their departure, and the onset of warm weather. Warm temperatures might result in snakes nesting earlier (e.g. earlier in June), but we searched for nests two weeks before their peak nesting period. All three of our main study areas did not experience any increase in development, habitat loss, or changes in road structure. Although all three are on protected land, they are well-known snake nesting areas, and poaching rates varied (presumably with interest and market values). Further, the use of ORVs has increased generally in the Pine Barrens, and this clearly impacted our study (see below). The increase in ORV use, and the disturbance ORV’s caused, may have influenced female choice of nest sites, and they might have moved away from the areas traditionally used.

With our increased experience came additional knowledge of Pine Snakes. Since we were more proficient at finding nests as time went on, the decrease in number of nests over time is real, and not due to our inability to find nests. Likewise, our ability to locate resting females during the nesting season increased. Our early studies in the 1970s and early 1980s involved branding snakes for identification. Our introduction of PIT tags in the late 1980s made identification easy and completely reliable. For 3 years we used both methods to convince ourselves that the PIT tags were effective in Pine Snakes, and that injecting the tags did not cause any harm, at the same time insuring that we identified all branded snakes correctly [46].

Some snakes were found in hibernacula in non-consecutive years, indicating that there are other hibernation sites within our study areas that our marked snakes used when we did not find them. Alternatively, when cold weather sets in, some snakes are far from hibernacula, and burrow down old stumps or roots solitarily (a few such snakes we located by radio-telemetry). We did not have continuous growth data on some snakes, although we did include these snakes in our survival estimates. For example, a snake may not have been found at 4 or 5-years, but if we found it at 8-years, it was clearly still surviving, and was included in the survival analysis. One male snake was located for several years in one hibernation area, and then moved to another 3-km away.

### Growth

Growth and survival are critical life history traits that directly influence fitness. A sufficient number of snakes need to reach sexual maturity and breed for a sufficient number of years to maintain the population. Not all individuals that reach sexual maturity contribute equally to the next generation. Growth is a key component of survival. Some species, such as snakes, have indeterminate growth, and individuals are expected to grow as quickly as possible to reach sexual maturity when there are no costs associated with rapid growth [52–54]. There may be costs to rapid growth, however, which leads to variations in size as a function of age [55], as well as age of sexual maturity. There is some evidence for decreased overwinter survival of reproductive females related to their emaciated state following parturition (Water Snakes [11]). Variations in growth can be caused by genotype, maternal condition, environment, colonization history, or a combination of these [4], and can be associated with latitude [6], gender [5], or human exploitation [56]. Growth rate can affect life-history traits such as size at maturity, reproductive output, and longevity [4, 57].

Growth rates in squamate reptiles are affected by prey availability under both field and laboratory conditions [58–60]. Growth rate and development can also be an effect of incubation temperature and habitat differences [9, 61, 62]. Garter Snakes (*Thamnophis elegans)* that had continuous access to prey and water exhibited fast growth, early maturation, high fecundity, and low adult survival, while those with variable prey exhibited slow growth, delayed maturity, low fecundity and high adult survival [16]. Presumably, these features are relative to one another, and not to Garter Snake populations in other geographical areas. On the other hand, prey availability early in life, leading to faster growth in the first year of life, can exert a long-term effect on growth trajectories and adult body size (the “silver spoon effect” [17]). For example, in Meadow Vipers (*Vipera ursiniii ursinii*), offspring in better condition grew faster than others, and heavier offspring had higher survival [2].

Sexual dimorphism in growth and size can be caused by factors intrinsic to the species, or to environmental factors (prey [16, 63]). In some snake species there is male biased sexual dimorphism, and in others the females are larger (Water Pythons *Liasis fuscus* [17]; Garter Snakes [5]). There is male-biased sexual dimorphism in Black Rat Snakes (*Elaphe obsolete* [7]), especially in older snakes. Still, in some other snakes there are no sex-related differences (e.g. Western Diamond-backed Rattlesnake, *Crotalus atrox* [24]; Bullsnake, *Pituaophis catenifer sayi* [6]). Such sexual dimorphism, however, is expected in fully adult snakes, and in the present study we only report data on the period up to reproductive maturity.

Examining growth is snakes is difficult because snakes are hard to find, are difficult to find at the same time each year, and it’s extremely difficult to estimate their ages (except for snakes marked from the hatchling stage, or in laboratory studies). In many field or natural studies, growth is determined by size-classes. That is, size classes were defined, and the rate of growth for snakes in that size class was determined. Thus aging snakes by size involves circularity (snake age is an assumption). Although defining age classes by size class was considered by Gibbons [64] to be a common method of aging individuals in natural populations of reptiles, he noted that using mark-recapture techniques (as in the present study) and following young of known ages is the best method. In our study, the age of all snakes reported in this paper is known because only snakes in the hatchling data set were considered.

Most studies locate, weigh, and measure snakes throughout their active season, and data are not from the same stage (or with respect to having a recent meal). Weight in snakes varies weekly and seasonally, depending upon the period since the snake ate, and reproductive stage (weight of gravid females varies markedly during the developmental period). In this study all snakes were weighed and measured at the same time (late Feb or early March), and all had been fasting since late fall, reducing variations due to season and eating patterns.

Pine Snakes in our study exhibited a similar growth pattern for males and females up to age 5-years, although as females aged there was greater individual variation in size. Since male-biased sexual dimorphism in size is often related to their engagement in combat competition for females [9], this may suggest a lack of such fighting over females in our Pine Snake population. Further, the relatively low population density of Pine Snakes in New Jersey [65] may mean that density is insufficient to result in competition among males.

Because Pine Snakes excavate their own nests and leave a characteristic dump pile, we can reliably locate nests, and often gravid females or females still in the nest. The reliability of being able to locate snake hibernacula, and locate gravid females and nests during the nesting season, allowed us to collect growth, age of first breeding, clutch size, and survival for a large sample of Pine Snake to determine recruitment.

### Age and survival to sexual maturity

Two of the key life history traits determining population viability is age of first breeding, and survival of eggs through hatching to reach sexual maturity (and reproductive activity). Both are difficult to determine for snakes, and are mainly determined from laboratory studies [59], or from field studies of hatchlings [1]. There may be a trade-off between early reproduction and body size; individuals that breed earlier may reproduce at a smaller body size than individuals with later maturity. Laboratory studies have shown that individuals on a low-energy diet grow more slowly, mature at a later age, and have smaller clutch sizes than females on a high energy diet (*Elaphe guttata* [59]). While laboratory studies can demonstrate what can occur, they do not necessarily reflect what does occur in nature.

One might predict that hatchling condition affects survival during the first year of life. For example, larger offspring had higher survival during the first year of life in Keelback Snakes (*Tropidonophis mairii*) incubated in a laboratory in Australia [62], and Garter Snake hatchlings (raised in laboratories) that were heavier at birth likewise survived better than others [4]. Body size was also positively related to survival in the Keelback Snakes mentioned above [62] and in Eastern Indigo Snakes (*Drymarchon couperi*) from Georgia [15].

However, in the present study of Pine Snakes, survival of hatchlings monitored until they were 4 and 5-years did not vary as a function of the length or weight of hatchlings. Instead, our data indicate that time period most influenced survival, with survival being highest in the early three periods (before 2000), compared to the latter periods (after 2000). Since these hatchlings were monitored once they reached hibernacula (having already survived the vulnerable neonate period), the survival rate was not influenced by poaching of hatchlings still in the nest, or emerging from their natal nests, but was due to mortality during years 1–3-years. The relatively higher mortality of young snakes (before age 4-years) implies greater vulnerability. It is possible that hatchlings in the period before 2000 grew faster, and some females may have attempted to breed (making them more vulnerable to predators and poachers on the nesting areas), but we do not have the breeding record for these snakes. Another possible explanation is that with the high level of ORV activity in the nesting habitat, poachers did not feel it was a productive area to search, other than during the nesting season. Lastly, it may be that survival in the early periods was normal, but survival in the period after 2000 was lower due to poor environmental conditions, fewer prey resources, or higher rates of predation.

Many studies of survival in snakes use size classes (not age classes) and mark-recapture models to estimate survival, and examine survival for all snakes (not males and females independently [15]). However, survival rates in our study of Pine Snakes relied on data from marked, known-sexed, known-aged individuals. There were sex-related differences in survival in the Pine Snakes in our study, even during the first 5-years of life. Males had higher survival rates than did females, even though there were no sex-related differences in growth rates (weights). There are differences in snake behavior that could affect our results. Male Pine Snakes move greater distances than females, and show lower fidelity to hibernation sites [25–28, 66]. Thus the actual survival of males at 4–5-years of age could be greater than our studies indicated. These individuals were followed for additional years, and if a snake was not encountered in the hibernaculum at age 4 or 5, but was located in a later year, it was considered alive for the purposes of the survival data. Female survival may also be underestimated if 4 or 5 year old females shifted nesting sites. Following the severe churning of nesting areas by ORVs during the 1990s, females may have returned to find that the ground where they last nested was loose “sugar sand” (soft), without vegetation or roots to stabilize their digging [32]. Some females may only have moved downslope to nest, but others may have left the traditional nesting area completely (and later entered a different hibernaculum that was not monitored).

Finding known age PIT-tagged individuals provides certainty, while not finding snakes causes uncertainty. Assumptions have to be made about survival based on not finding a snake for a certain number of years. For example, individual Black Rat Snakes were considered dead if not captured within 2-years after initial capture [7]. However, in our study, a snake could be missing (e.g. not found in a monitored hibernacula) for many years, but still be found later. For example, although unusual, one male (PIT tagged as a hatchling) was located at age 3-years, and then not again until it was 17-years old, when it returned to the same hibernaculum (Burger and Zappalorti, Unpub. Data). Another PIT-tagged male was not located between the ages of 7 and 23, while in contrast a female was located 19 out of 23 times in the same hibernaculum. These records for males, however, are unusual. Additionally, radio-tracked snakes revealed significant mortality by hawk predation and ORVs [19].

Information on age of first breeding is also critical for understanding life histories and population stability [22]. Pine Snakes are considered late-maturing temperate colubrids [14]. Little was known of the age of first breeding in Northern Pine Snakes or their close relatives, although Imler [67] reported that Bullsnake (was then *Pituophis melanoleucus*, now *Pituophis catenifer sayi)* bred at 3-years of age. Parker and Brown [68] reported that males matured at snout-vent lengths of 96-cm (range of 67–125) and females matured at 98.5 (range of 78–114) for Gopher Snake in Northern Utah (now *P*. *catenifer* [69]). Pine Snakes, however, grow to a longer maximum length, and it is likely that they may not breed as early.

Our information on snout-vent length and clutch size in Pine Snakes reflects field conditions, based on capturing gravid or laying females, and indicates that female Pine Snakes are capable of laying eggs at snout-vent lengths of 90-cm greater (Table 5). Using this measure, we determined that some 3-year old females may breed, all but one 4-year old female likely breed, and all 5-year old females (dotted line on Fig 1) could theoretically breed. Presumably, however, body condition (i.e. weight and prey availability) influences whether they do breed, which requires following known-age females to the nesting areas. In the present study, snout-vent length was highly correlated with weight at the end of hibernation (e.g. tau = 0.90). However, by 4–5-years of age, there was more variability in weight in females than in males (refer back to Fig 3). Thus, 3 or 4-year old females at the lowest weights may be less likely to breed, or may lay fewer eggs (clutch size), than heavier females.

### Sex ratios

Sex ratios in snakes are assumed to be close to unity at hatching [14]. Snakes do not have temperature influenced sex determination. In laboratory studies we found that although sex ratio (male—female) was not determined by incubation temperature, male embryos suffered greater mortality at lower incubation temperatures [47], resulting in a female-biased sex ratio. At 30^o^ C (one of the highest incubation temperature used in the laboratory, and the highest recorded in the field), sex ratio was male-biased.

Sex ratios of snakes caught in the wild are very dependent upon time of capture; males are more detectable in spring when they are searching for females, and females are more detectable during the nesting season [14]. We reported that sex ratios of snakes caught in the field (e.g. not in hibernacula) in the late 1970s to the mid-1980s varied by snout-vent length, and season of location, and was always female-biased. Sex ratio varied from 0.78 M: F in hatchlings, to 0.40 M: F in the largest snakes [47]. Thus, simply reporting sex ratios without regard to season (or activity) does not account for imperfect detection, or may rely on assumptions of an equal probability of males and females being present and does not address the differences due to sampling time. Mazerolle et al. [70] provide some methods to reduce this bias, including the traditional Lincoln-Peterson Index for closed populations, or the Jolly-Seber model for open-populations (more similar to our approach).

Sex ratios of Pine Snake hatchlings in the present study were determined by examining snakes while hibernating. Overall (1986–2015), 41% of hatchlings in hibernacula were males. (sex ratio = 0.69 M:F), but when known-age 2-year olds were added, the sex ratio was 0.59 (M:F, see Table 2). For these known-aged snakes, survival was higher for males than females, leading to an increased sex ratio for 4 (0.79) and 5 (0.72) year olds. Older Pine Snakes may show a male-biased sex ratio since females are preferred by poachers because they can breed them and sell the offspring, and females are much easier to catch while nesting [19]. Further, as shown for other snakes, there may be some female mortality overwinter due to emaciation because of egg laying [11]. We never found any dead adults in hibernacula (except those that froze because they were near the surface). However, the cost of egg-laying may take its toll in terms of body condition (e.g. weight). There is some indication that a few older (and longer) females had relatively low weights for their body length (refer to Fig 3). If females have higher mortality than males due to the costs of egg-laying (due to body condition, and to poachers), then sex ratios should continue to shift with age of the snakes.

### Survival, recruitment to breeding age, and human intervention

Pine Snakes living in the New Jersey Pine Barrens require suitable habitat for foraging, resting, nesting, and hibernating. They also require habitat that is relatively free from human intervention. Human intervention can come in the form of habitat destruction due to development, or management to prevent disturbance and destruction. Disturbance and destruction includes the slow (or rapid) degradation and loss of habitat, and the more immediate destruction of nesting habitat by ORVs, as well as poaching of eggs and hatchlings. Management can include habitat protection or improvements (e.g. removal of saplings, herbs and grasses that invade nesting sites), or prevention of poaching and exclusion of ORVs. The nesting areas in Ocean and Burlington County were prime for the development of senior communities, and over the 1980–2015 period, the population grew 71% (Ocean) and 24% (Burlington) counties (U.S. Census Bureau web site).

Nesting areas need to be relatively open to provide sun-penetration to the ground (to heat the eggs): otherwise there is lower hatching success, incubating takes longer, hatchlings are born with morphological and behavioral abnormalities, and at the lowest incubation temperatures (found in some nests in the Pine Barrens), hatchlings do not have sufficient time to hatch, forage, and reach a hibernaculum before the cold weather sets in [43, 48, 61, 71–73]. Similarly, hibernacula are usually at the edges of clearing where they are also exposed to sun penetration [18]. Open patches that are ideal for Pine Snake nesting were originally created by Native Americans (who burned parts of the Pine Barrens to increase available deer), later fires were started by sparks from coal burning locomotives along railroads, and still later fires were set by European settlers who eked out a living on small farms carved in the pines [18]. Farmers often left small areas at the edges of fields for the Pine Snakes to nest (they appreciated the snakes for keeping down the rodents that ate their crops). Many of these clearings have succeeded to pine forest with a closed canopy, reducing the number of available nesting sites [18, 65].

Pine Barrens habitats that are far from sand or paved roads are generally more pristine and undisturbed, particularly if the sand roads have deep sugar sand making it difficult for cars to use. If nesting clearings are available, and the forest surrounding the clearings provides adequate foraging conditions, Pine Snake populations can flourish. More often, however, clearings are disappearing. Active fire prevention and suppression on NJ State Forest lands is unfavorable for Pine Snake habitat because it prevents the creation of open patches, and is also invoked as a threat to the habitat necessary for Black Pine Snakes [74] and Louisiana Pine Snakes [75].

The Pine Snake populations we study nest in relative pristine, protected areas of the Pine Barrens, but this does not prevent them from facing intense and direct human pressure from ORVs and poachers. New Jersey Pine Snakes are highly prized in the pet trade because they have a distinct black and white pattern that other Pine Snakes to the south do not have. For instance, the Florida Pine Snakes tend to have brown or tan blotch patterns and some lack black or brown altogether and appear light tan color. As their common name implies, Black Pine Snakes almost lack the white ground coloration altogether, making the snakes largely black in populations to the south [18, 76]. The pressure from poachers is increasing on our study sites, largely because other, more accessible nesting areas near roads have already been over collected and gravid females and eggs in nests have all been removed. Because of Internet posting of good collecting areas and Google Earth maps, our traditional study areas have been found by poachers and are well-known. Even though our study sites are difficult to reach on unimproved sand roads many people now have four-wheel drive vehicles, so they can access the site easily. These remote nesting areas are removed from the eyes of police and conservation officers. Even if conservation officers see a parked car and walk toward the nesting area, poachers either have a look-out or they hear the car approaching, and can quickly disappear into the pine forest where they stash their snake bags, and emerge innocently with no evidence that they are collecting snakes.

The threat of poaching is difficult to solve in remote situations where the Pine Snakes are visible and vulnerable during the nesting season, and the females leave the nest exposed. Recently, the Endangered and Nongame Species Program of New Jersey Department of Environmental Protection started using camera surveillance over our nesting areas. Once the surveillance is known, poachers may avoid the area. The cost of personnel and surveillance cameras makes this less than ideal, however. Poaching has continued, and we have again started a “head start” program of collecting egg clutches before the poachers can steal them. We now hatch the eggs in the laboratory, and place the neonates back in their natal nests in the early fall, allowing them to emerge on their own. Our experience of observing neonate Pine Snakes emerging from nests has shown that they can “disappear” into the Pennsylvania Sedge (*Carex pensylvanica*), heather ((*Hudsonia* sp.) or pine needles, suggesting that they have a low vulnerability to poachers, but perhaps a high vulnerability to mammal and bird predators (Burger and Zappalorti, Unpub. Data). Poachers primarily target females during the nesting season, although they will take any snake they find. In general, protecting adult snakes and their habitats results in the highest likelihood of long-term population stability [15], corroborating the importance of protecting the nesting females.

The second, major cause of decreased reproductive success was ORVs. Those who drive ORV’s seek all kinds of open terrain and mounds to drive over. In the 1990s, ORV drivers discovered some nesting areas; one of our sites proved ideal from their perspective. It is a large area with few trees, and a slope making ORV riding challenging. In some years several ORVs raced around the site, making deep ruts in the sand where the snakes traditionally nested. These sites are still not completely suitable for nesting, even 16-years after a very high berm and thick steel cables were installed to block the sand road entrance. Many more years will be required for the delicate heather, sand wort *(Arenaria caroliniana*.) and low grassy vegetation to recolonize these areas. Unfortunately, early successional stage colonizers, such as switch grass (*Panicum virgatum*), broom sedge (*Andropogon virginicum*), shrubs, and sapling trees, have moved in and require manual removal to allow full sun penetration to the sand surface. Without nearly full sun exposure, ground surface temperatures tend to be lower (cooler), and eggs take longer to hatch or do not hatch at all. If they do hatch late, neonates hatch with behavioral and morphological abnormalities (see above). Wind, birds, mammals and ORVs likely carry in the seeds of unwanted plant species in their droppings, hoofs, or between tire-treads, which are additional impediments to maintaining open, sunny nesting areas that are selected by gravid female Pine Snakes.

Although not addressed in this paper, ORVs kill snakes as they move about in their habitat by purposely or accidently running them over during the spring, summer and fall. Pine Snakes cross roads when foraging, seeking mates or during spring or fall migrations to and from winter dens. These are peak times when all-terrain vehicles drive on sand trails through the pine forest, and run over the snakes. They are particularly vulnerable when they first leave hibernacula because they are still cold, move slowly, and often bask on the more-open bike trails. The snakes killed by motorcycles are difficult to find because they are rapidly eaten by predators. One of our 12-15-year old female Pine Snakes was killed in March 2016 by an all-terrain bike (only discovered because she had a radio-transmitter).

Preservation of viable Pine Snake populations requires that sufficient males and females survive to reach sexual maturity and successfully breed for enough years to replace themselves. Our 30 + years of data indicate that if eggs in nests hatch and a high percentage of hatchlings reach a hibernacula, then recruitment for that season is successful. However, the percent of neonate survival varies each year and is partly dependent upon human activities. Poaching and ORVs use must be managed to ensure that sufficient Pine Snake hatchlings reach sexual maturity. Conservation solutions include camera surveillance of known important nesting areas, barriers to prevent ORV movement onto nesting and denning areas, checking pet shops and reptile shows for Pine Snakes with PIT tags, stiffer regulations (and penalties) for illegal collecting and ORV use in critical nesting and denning areas, especially enforced during periods of ingress and egress from hibernacula, and expanded anti-poaching enforcement. Meanwhile, continued “head start” programs (e.g., hatching eggs in the lab and releasing neonates back to the nest site), can reduce the effects of predation and poaching on nest success. Regular monitoring of nest success is needed to ensure management works and to allow adaptive management. Education of the public to appreciate the ecological role of Pine Snakes in the environment is critical. Citizens should report poachers to conservation officers via the NJDEP hot line, which will discourage illegal collecting. Poachers operate in remote Pine Barrens areas, so citizens should take pictures of license plates and report poaching to the NJDEP, rather than confronting them. The New Jersey Pine Barrens, although at the northern limit of their range, is nonetheless the center of Northern Pine Snake populations [18, 65], and maintaining healthy populations in New Jersey is critical to the species survival.

## Supporting information

S1 Table Growth of males and females (for Table 3). (XLSX)
